# Reducing intrusive memories after trauma via a brief cognitive task intervention in the hospital emergency department: an exploratory pilot randomised controlled trial

**DOI:** 10.1038/s41398-020-01124-6

**Published:** 2021-01-11

**Authors:** Marie Kanstrup, Laura Singh, Katarina E. Göransson, Julia Widoff, Rod S. Taylor, Beau Gamble, Lalitha Iyadurai, Michelle L. Moulds, Emily A. Holmes

**Affiliations:** 1 Division of Psychology, Department of Clinical Neuroscience, Karolinska Institutet, Stockholm, Sweden; 2 Functional Area Medical Psychology, Karolinska University Hospital, Stockholm, Sweden; 3 Department of Psychology, Uppsala University, Uppsala, Sweden; 4 Emergency and Reparative Medicine Theme, Karolinska University Hospital, Stockholm, Sweden; 5 Department of Medicine Solna, Karolinska Institutet, Stockholm, Sweden; 6 MRC/CSO Social and Public Health Sciences Unit & Robertson Centre for Biostatistics, Institute of Health and Well Being, University of Glasgow, Glasgow, UK; 7 Department of Psychiatry, University of Oxford, Oxford, UK; 8 School of Psychology, The University of New South Wales, UNSW Sydney, Sydney, Australia

**Keywords:** Psychiatric disorders, Human behaviour

## Abstract

Intrusive memories are common after trauma, and can cause significant distress. Interventions to prevent/reduce the occurrence of this core clinical feature of posttraumatic stress disorder are needed; they should be easy to deliver, readily disseminated and scalable. A novel one-session intervention by Iyadurai et al. 2018, *Molecular Psychiatry*, resulted in intrusion reduction over the subsequent week. Its feasibility in a different setting and longer-term effects (>1 month) need investigation. We conducted an exploratory open-label pilot randomised controlled trial (RCT) to investigate the feasibility and effects of a brief behavioural intervention to reduce intrusive memories in trauma-exposed patients in a Swedish hospital emergency department (ED). Participants (final *N* = 41) were randomly allocated to either intervention (including memory reminder cue then visuospatial cognitive task “Tetris” with mental rotation instructions) or active control (podcast) condition within 72 h of presenting to the ED (both conditions using their smartphone). Findings were examined descriptively. We estimated between-group effect sizes for the number of intrusive memories post-intervention at week 1 (primary outcome) and week 5 (secondary outcome). Compared to the control condition, participants in the intervention condition reported fewer intrusive memories of trauma, both at week 1 and week 5. Findings extend the previous evaluation in the UK. The intervention was readily implemented in a different international context, with a mixed trauma sample, with treatment gains maintained at 1 month and associated with some functional improvements. Findings inform future trials to evaluate the capacity of the cognitive task intervention to reduce the occurrence of intrusive memories after traumatic events.

## Introduction

Intrusive memories of traumatic events are commonly experienced in the aftermath of psychological trauma. They come to mind involuntarily, are repetitive, and can elicit significant distress and impair functioning. Intrusive memories are the cardinal symptom of posttraumatic stress disorder (PTSD)^1–3^. These sensory memories typically comprise visual mental imagery^4^ from moments in the trauma^5^. Intrusions in the acute post-trauma phase have been centrally associated with other acute posttraumatic stress symptoms^6^, and the intrusion symptom cluster is associated with the longitudinal course of PTSD^7^. Interventions to target intrusive memories in the acute phase may help because intrusions can be distressing in their own right^1^. Moreover, it is possible that early intrusions could serve as a ‘clinical marker’^8^ for persistent intrusions and PTSD^9^.

To have meaningful impact given the global scale of trauma, it would be helpful to have interventions that are simple to administer and accessible outside traditional psychotherapy settings, e.g., in hospitals (following medical trauma), or community settings (e.g., after major accidents or terror attacks), as well as scalable (e.g., for trauma-exposed individuals who are refugees or vulnerable groups during a pandemic). Current evidence-based psychological treatments after trauma include numerous components to address the breadth of symptom clusters which make up the PTSD diagnosis. Meanwhile NICE guidelines for PTSD^10,11^ now consider interventions targeted at *specific symptoms* in some circumstances such as when other interventions are not available. Holmes and colleagues developed a preventative approach targeting just one core clinical feature^12^ after trauma^13^. This novel, brief behavioural intervention to reduce the number of intrusive memories soon after trauma draws on ideas from cognitive science (cognitive task interference^14^/memory (re)consolidation^1,15,16^) rather than traditional exposure models^17–19^.

The cognitive task intervention comprises several parts including a brief memory reminder/orientation (to re-/activate specific trauma memory ‘hotspots’^5^), followed by a visuospatial cognitive interference task (e.g., playing computer game ‘Tetris’ alongside training to engage in ‘mental rotation’ throughout), administered according to specific timings and order. This task is theorised to interfere with (re)consolidation of visuospatial components of the trauma memory, targeted at those memory segments that intrude (hotspots). Laboratory research using experimental trauma^20^ demonstrated that the intervention reduces the number of intrusions over the subsequent week compared to control conditions^21–25^.

Moving from the laboratory to clinical settings with individuals exposed to real trauma, accruing preliminary evidence suggests the intervention may reduce the occurrence of intrusive memories both when recently acquired^3,26^ and longstanding (consolidated)^27–29^. For example, women who completed the intervention soon after traumatic childbirth (6 h after an emergency caesarean section in a Swiss hospital) reported fewer intrusive memories (by 48%) in the subsequent week relative to the (usual care) control condition^26^. For psychiatric inpatients (in Germany) with complex PTSD and longstanding intrusive trauma memories, those intrusions targeted by the intervention (procedure adapted for older memories) reduced by 64% from pre- to post-intervention compared to a reduction of 11% for non-targeted intrusions^27^.

Iyadurai et al.^3^ reported that motor vehicle accident survivors in a UK hospital emergency department (ED) who received the intervention (delivered by a clinical psychologist) up to 6 h post-accident reported fewer intrusions over the subsequent week (by 62%) relative to participants in an active control (activity log task) condition (*d* = 0.67, 95% CI: 0.18, 1.14)^3^. There were convergent findings on a clinical measure of distress related to posttraumatic stress intrusion symptoms at 1 week (IES-R^30^), but not for other symptom clusters. The primary outcome (intrusive memory diary) was administered at 1 week (as in earlier laboratory studies), but not re-administered after 1 month. No between-group differences were found at 1 month (see Iyadurai et al.^3^, Table 1). The authors noted that the study was designed to detect an effect on the primary outcome measure at 1 week (diary), thus there may have been insufficient power to test secondary hypotheses, warranting a larger trial powered to detect differences at 1 month.

**Table 1:** Questionnaires at each timepoint.

	Day 1 (Baseline)	Week 1	1 month	Week 5	3 months	6 months
*Emergency department*						
Demographics	X					
Psychological/medical/ trauma history	X					
Perceived threat	X					
PDEQ	X					
PDI	X					
Credibility/Expectancy Questionnaire	X					
SRHR	X	X	X		X	X
SRSR	X	X	X		X	X
*After discharge*						
Intrusive memory diary		X		X		
IES-R		X	X		X	X
HADS		X	X		X	X
PSS			X			
WSAS		X	X		X	X
Feedback Questionnaire about participation		X	X		X	X
Sensory modality of intrusive memories		X	X			
MINI section H^a^			X		X	X

*IES-R* Impact of Event Scale–Revised, *HADS* Hospital Anxiety and Depression Scale, *WSAS* Work and Social Adjustment Scale, *SRHR* Self Rated Health Rating, *SRSR* Self-Rated Sleep Ratings, *PDEQ* Peritraumatic Dissociative Experiences Questionnaire, *PDI* Peritraumatic Distress Inventory, *PSS* Perceived Stress Scale, *MINI* The Mini International Neuropsychiatric Interview.

^a^Completed via telephone.

Implementation feedback and information on adverse events was also collected (see Materials a and Methods).

A further limitation of Iyadurai et al.’s^3^ study is that the intervention and control conditions differed in the way in which they were delivered (intervention task on a game console, control task using pen-and-paper). Moreover, all participants were survivors of a motor vehicle accident, leaving untested the question of whether findings extend to a mixed trauma sample. Follow-up trials to Iyadurai et al.’s^3^ preliminary investigation are therefore needed.

The main aim of the current pilot randomised controlled trial (RCT) was to investigate the effects of the simple cognitive task intervention on intrusive memories, and other symptoms, after a traumatic event, with follow-ups at both 1 week and 1 month, and where possible 3 and 6 months. The intervention task was delivered by smartphone (rather than game console) and more closely matched to the control condition (a podcast, also via smartphone)^31,32^. We employed a more diverse trauma sample not limited to motor vehicle accident survivors. We examined whether the intervention could be effectively implemented in a new hospital and international context (Sweden), following initial feasibility work^33^. The aim of the present exploratory pilot study was to present descriptive results, rather than a test of statistical significance. We conducted planned analyses to obtain an estimate of effect size of the difference between conditions on the number of intrusive memories (diary). Such information can guide the design, including power and sample size estimation, of future follow-up RCTs.

As noted, we used the intrusive memory diary to monitor intrusions in week 1 post-trauma in previous work^3,26^, but not beyond. We were interested in the feasibility of also administering it at week 5 to inform its potential as an outcome measure in future trials. Participants therefore monitored their intrusive memories in the diary in two separate weeks (week 1 and week 5). The Clinical Trial Registration (NCT03509792) lists number of intrusive memories at ‘one month’ (rather than at week 5) as the secondary outcome. For the sake of clarity, we have opted to refer to this measure as taken at week 5, to specify the timing of the diary measure over a full week (during week 5) in relation to the administration of the intervention/control task (day 1). The intervention was delivered by students (Psychology MSc) rather than qualified mental health professionals. For such implementation, each team member received several training sessions, following a formal training protocol including role-play, feedback and observation of initial cases. Finally, we included additional measures of functional impairment, to more thoroughly gauge the extent to which any reductions in intrusive memories were associated with functional gains (e.g., sleep, concentration).

In summary, this pilot RCT aimed to investigate the feasibility and effects of a cognitive task intervention versus active control on the number of intrusive memories of trauma at week 1 (primary outcome) and week 5 (secondary outcome) as well as other symptoms (post-traumatic stress, anxiety, depression) and functioning at 1 week and 1, 3 and 6 months.

## Materials and methods

### Participants

**Fig. 1:**
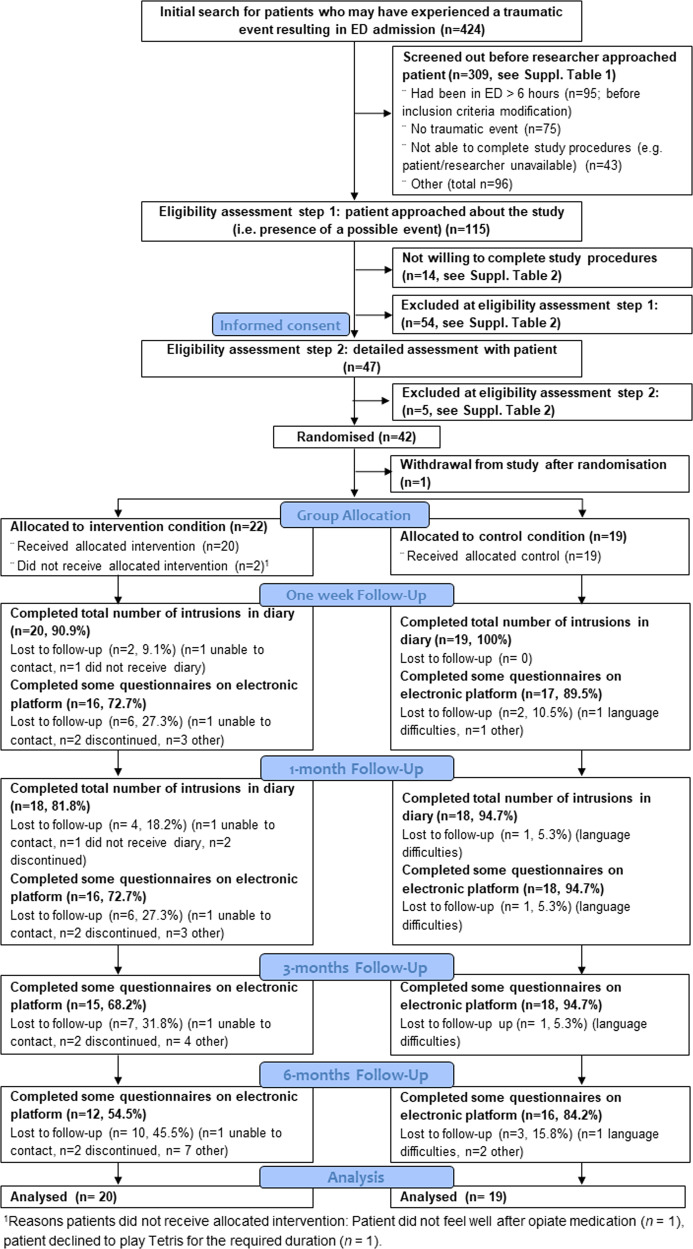
Participant flow through the study. CONSORT diagram.

We aimed to recruit ca. 40 participants, a feasible sample size for this exploratory pilot study. Participants (*N* = 42) were recruited following presentation to the ED of Karolinska University Hospital Huddinge, Stockholm, Sweden (*n* = 37), and the nearby ED Walk-in Centre (*n* = 5). One participant withdrew from the study and their data are not included; the final sample therefore included 41 participants (23 female), see CONSORT participant flow diagram, Fig. 1 (and Supplementary Tables 1 and 2 for details). Mean age was 46.15 years (SD = 15.77), see Table 2 for details.

**Table 2:** Participant characteristics per condition.

	Intervention (*n* = 22)		Control (*n* = 19)	
	*Mean* *(Range)*	*SD*	*Mean* *(Range)*	*SD*
Age (years)	45.14 (19–66)	14.51	47.32 (20–76)	17.45
	*n*	%	*n*	%
*Gender*				
Female	14	63.6	9	47.4
Male	8	36.4	10	52.6
Other	0	0	0	0
*Highest level of education*				
Elementary school	1	4.5	0	0
Upper secondary school	8	36.4	8	42.1
University	13	59.1	10	52.6
Other (no school education)	0	0	1	5.3
*Place of birth*				
Sweden	17	77.3	14	73.7
Other Scandinavian country	1	4.5	0	0
Other European country	0	0	1	5.3
Outside of Europe	4	18.2	4	21.1
*Marital status*				
Single	4	18.2	5	26.3
Married or cohabiting	12	54.5	13	68.4
Divorced	1	4.5	0	0
Widowed	0	0	0	0
Living apart together (LAT)	5	22.7	1	5.3
Other	0	0	0	0
*Employment status*				
Employed	22	100	16	78.9
(Full-time employed)	(19)	(86.4)	(13)	(68.4)
(Part-time employed)	(3)	(13.6)	(2)	(10.5)
Unemployed	0	0	1	5.3
Student	0	0	0	0
Retired	0	0	3	15.8
On sick leave	0	0	0	0
Other	0	0	0	0
*Yearly income (SEK)*				
0–249,999	5	22.7	4	21.1
250,000–349,999	4	18.2	6	31.6
350,000–449,999	7	31.8	4	21.2
450,000–549,999	4	18.2	2	10.5
550,000 and above	2	9.1	3	15.8
	*n*	%	*n*	%
*Traumatic event*				
DSM-5 PTSD criterion A1	22	100	19	100
Experienced event	22	100	19	100
Witnessed event	0	0	0	0
Brought in by ambulance	4	18.2	8	42.1
Type of trauma^a^				
Transportation accident	8	36.4	7	36.8
(Car/van/bus driver)	(5)	(22.7)	(2)	(10.5)
(Car/van passenger)	(0)	(0)	(1)	(5.3)
(Motorcyclist)	(0)	(0)	(1)	(5.3)
(Electric scooter driver)	(0)	(0)	(2)	(10.5)
(Cyclist)	(3)	(13.6)	(1)	(5.3)
(Pedestrian)	(0)	(0)	(0)	(0)
Serious accident at work, home, or during recreational activity	13	59.1	11	57.9
(Slip-and-fall injury)	(6)	(27.3)	(6)	(31.6)
(Free fall trauma)	(2)	(9.1)	(3)	(15.8)
(Threat to limb/extremity)	(2)	(9.1)	(1)	(5.3)
(Head injury)	(1)	(4.5)	(0)	(0)
(Burn injury)	(1)	(4.5)	(0)	(0)
(Attacked by dog)	(1)	(4.5)	(0)	(0)
(Crushed under heavy object)	(0)	(0)	(1)	(5.3)
Assault with a weapon	1	4.5	0	0
Physical assault	0	0	1	5.3
	*n*	%	*n*	%
Perceived life/serious injury threat to self (score > 0)	18	81.8	16	84.2
Perceived life/serious injury threat to someone else (score > 0)	2	9.1	5	26.3
	*Mean*	*SD*	*Mean*	*SD*
Perceived threat	5.64	3.17	5.37	3.29
Time since traumatic event (hours:min)				
Included within 6 h since traumatic event (intervention *n* = 14, control *n* = 17^b^)	2:33	0:58	3:21	1:13
Included within 72 h since traumatic event (intervention *n* = 8, control *n* = 2)	25:10	14:37	12:37	6:52
Injury Severity Score	2.05	1.68	2.16	1.61
PDEQ	16.23	7.96	19.16	9.22
PDI	14.18	8.78	17.32	9.26
	*n*	%	*n*	%
*Treatment in emergency department*				
Location in emergency department				
ED	20	90.9	16	84.2
ED Walk-in centre^c^	2	9.1	3	15.8
Admitted as in-patient	2	9.1	1	5.3
Received opiate medication	8	36.4	10	52.6
	*n*	%	*n*	%
*History of trauma or mental illness*				
Prior psychological trauma	17	77.3	16	84.2
Current/past mental illness	6	27.3	6	31.6
Family history of mental illness	8	36.4	5	26.3
	*Mean*	*SD*	*Mean*	*SD*
*Self-rated sleep and health (baseline)*				
SRHR	5.82	1.10	5.37	1.21
SRSR	5.91	2.35	5.00	2.98

*DSM-5* Diagnostic and Statistical Manual of Mental Disorders, fifth edition, *PDEQ* Peritraumatic Dissociative Experiences Questionnaire, *PDI* Peritraumatic Distress Inventory, *SEK* Swedish crown, *SRHR* Self-Rated Health Rating, *SRSR* Self-Rated Sleep Rating.

^a^Classified by the Life Events Checklist LEC-5.

^b^Note that one participant was included within 6 h but completed the task 6–72 h since traumatic event.

^c^Walk-in centre only included as a recruitment site from 3 June 2019 onwards.

Inclusion criteria were: aged over 18 years, experienced a Criterion A traumatic event (DSM-5^2^); e.g., motor vehicle accident, industrial accident, assault) resulting in ED admission, reported memory of the trauma, fluent in Swedish, alert and orientated, access to a smartphone and sufficient physical mobility to use it. In the initial trial protocol, participants were eligible for inclusion if seen in the ED within 6 h of the trauma. However, to increase recruitment rate and given findings showing that the intervention can be used 72 h after analogue trauma^34^, the protocol was modified (on 8/5/2019, after 18 participants, see *Procedural Changes* in *Supplementary Information*) and the timeframe changed to within 72 h (which comprised *n* = 11/41; 8 in intervention). Exclusion criteria were: loss of consciousness of >5 min, history of severe mental illness, current intoxication, substance abuse/neurological condition, current suicidality. Consecutive sampling was used. Of 115 patients approached, 14 were not willing to participate and 59 were not eligible (Fig. 1).

### Measures and materials

#### Information about the traumatic event

##### ‘Hotspots’ sheet

Participants in the intervention group were asked to briefly mention their worst moment images of the traumatic event that led to their arrival in the ED (e.g., ‘the truck is coming toward me’). These were listed on a sheet of paper and used as part of the intervention procedure so that the memory hotspots were held in mind prior to the visuospatial interference task, as in our previous work^3^.

#### Primary and secondary outcome measures

##### Intrusive memory diary

Participants recorded intrusive memories in a pen-and-paper diary^3,26^. Instructions included a definition of intrusive memories (i.e., visual memories of moments from the trauma that come to mind unbidden, not deliberately recalled, not verbal thoughts), and details about how to record their occurrence by marking a box at the time of day (morning, afternoon, evening, night) each intrusion was experienced. If no memories were experienced, participants were asked to indicate this by writing zero. For the primary outcome, i.e., number of intrusive memories in week 1, participants completed the diary for 7 days, starting after the intervention/control task (day 1). Researchers sent daily SMS reminders to complete the diary. At the end of the week, participants returned the diary by post.

Participants completed a second identical 7-day diary, commencing 1 month after the intervention/control task (week 5; i.e. day 28; secondary outcome).

On the final day of each monitoring week (weeks 1 and 5), participants rated diary accuracy (*‘How accurately do you think you completed the diary?’*, 0 = not at all, 10 = extremely).

##### Impact of Event Scale–Revised (IES-R)

This 22-item scale assesses subjective distress after trauma on three subscales: intrusion symptoms, avoidance and hyperarousal^30^.

##### Hospital Anxiety and Depression Scale (HADS)

Two 7-item subscales assessing symptoms of anxiety and depression^35^.

#### Other pre-specified outcome measures

##### Credibility/Expectancy Questionnaire

Before completing the assigned condition (day 1), participants provided five ratings of treatment expectancy and the degree to which they found the rationale for treatment credible (11-point scale, 0 = not at all, 10 = extremely), adapted for the current study^36^. Total score ranges from 0–50; higher scores indicate more credibility.

##### Self Rated Health rating (SRHR)

A single item with a 7-point scale assessing perceived health status from very good to very bad. Higher scores indicate better health^37^.

##### Self Rated Sleep ratings (SRSR)

Assesses extent of sleep problems using a 5-point scale (not at all to very much, reverse scored) and reported number of nights per week with sleep problems (from 0–1 to 5–7 nights)^38^. Higher values indicate better sleep.

##### Adverse events

During each follow-up call, participants were asked whether they had experienced any adverse events. Any reported to research assistants were reviewed by the PI (EAH) to evaluate seriousness and relation to study procedures.

##### Characteristics of intrusive trauma memories

Self-rated bespoke items assessing the degree of intrusion-related vividness, distress (both 0 = not at all, 10 = extremely), and concentration disruption (1 = not at all, 9 = very).

#### Apps for intervention and control tasks

##### Tetris smartphone app

The computer game Tetris^39^, a visuospatially demanding game, was downloaded on intervention group participants’ smartphones. The app version used was available for both Android and iOS devices, and contained an official Tetris game with the options to select ‘ghost piece off’ and play using ‘marathon mode’; both settings were selected prior to gameplay. Sound was turned off. The game involves participants moving seven differently shaped blocks into horizontal lines, as they appear on the screen. Participants were instructed to engage in ‘mental rotation’ as they played (see section “Treatment conditions: Intervention”).

##### Swedish Radio (SR) app

The SR app^40^ was downloaded on control group participants’ smartphones. They were instructed to find the program ‘Filosofiska rummet’^41^, and listen to it using their headset (or disposable study headsets).

### Treatment conditions

Participants undertook study procedures during their time in the ED. Care was taken to ensure that all steps of study participation fitted into wait-times and did not affect medical care provided in the ED. Routine hospital procedures for hygiene and safety were followed. Data collection was conducted by trained research assistants (JW, OK, FS, YW). Study procedures followed a written structured protocol based on previous work^3^.

#### Intervention

The behavioural intervention comprised a cognitive task procedure including a brief memory reminder procedure (so that the individual’s hotspots were held in mind) prior to engaging in a visuospatial interference task—playing the computer game Tetris—with specific instructions for ‘mental rotation’. The memory reminder procedure consisted of completing the ‘hotspots’ sheet; i.e., participants briefly named their worst moment images within the traumatic event and listed them on a sheet of paper. This sheet remained in front of the participant for the duration of the intervention. For the gameplay component, participants were instructed to actively use ‘mental rotation’; i.e., visualise ‘in their mind’s eye’ how to rotate/move the Tetris blocks to fit them into horizontal lines—planning ahead for the next few blocks to appear on the screen. After downloading the app and receiving instructions, participants practiced with the researcher present and then played the game using mental rotation instructions for at least one uninterrupted period of 10 min, and for ~20 min in total. Participants were given the option of engaging in self-administered booster sessions after their session in the ED, i.e., play the game in a similar way at home or during daily life if/when intrusive memories occurred. When we revised inclusion criteria, the intervention procedure was adapted accordingly (see *Procedural Changes* in *Supplementary Information*).

#### Active control

Control group participants listened to a podcast via the SR app^41^. Following instructions on how to download and open the podcast, participants listened on their smartphone for at least one uninterrupted period of 10 min and for ~20 min in total (see *Procedural Changes* in *Supplementary Information*).

In both conditions, researchers remained nearby to observe and encourage participants to engage in the task (gameplay or podcast).

#### Training to deliver the intervention

To promote protocol fidelity and appropriate delivery in a hospital setting, it was critical that individuals administering the intervention and control received adequate training, feedback and monitoring. Based on prior feasibility work in Sweden^33^, we developed formalised training for research assistants to complete prior to delivering the study protocol. Training took place in a group (approximately four students, trained by two experienced clinical psychologists/researchers). Training material (presented in PowerPoint) covered key learning points for all steps of the protocol and included a checklist for trainers and trainees to monitor individual progress.

Training included (1) a half-day introductory course for Good Clinical Practice; (2) approximately 2 days of initial training comprising key knowledge required to deliver the protocol (i.e., ethical considerations in trauma research, communication skills with trauma patients, how to understand and focus on the crucial parts of the intervention—identifying hotspots, mental rotation instructions and 20 min of gameplay), roleplay of crucial parts of the protocol, e.g., (i) taking informed consent, (ii) delivering the intervention, (iii) helping participants understand/complete the intrusive memory diary; (3) reflective group feedback by experienced clinical psychologists on 1 and 2 above, close observation and individual feedback by the supervising clinical psychologists in real-time during recruitment of the first at least three participants; (4) continued real-time supervision via phone/video call regarding inclusion/exclusion criteria, procedural concerns in the ED and intervention delivery as needed; (5) intermittent observations to support adherence to protocol; (6) regular fidelity checks and feedback regarding incoming data acquisition.

### Procedure

Trained research assistants identified potential participants in the ED in collaboration with ED staff. Participants deemed eligible (assessed in consultation with supervising clinical psychologists [EAH, MK] and ED staff) were given further oral and written information about the study. After providing written and informed consent, participants completed baseline assessments, including demographic information and details about the traumatic event that brought them to the ED (*Supplementary Information*).

They were then randomly allocated in a 1:1 ratio to two parallel treatment conditions using blocked randomisation (block size = 4) performed by a clinical trials unit (Karolinska Trial Alliance) with a web-based randomisation system (from http://www.randomization.com/). In line with Iyadurai et al.^3^, randomisation was carried out using stratification based on perceived threat to self/other. The sequentially numbered randomisation envelopes were stored and opened away from participants, and accessed by research assistants after baseline measures had been completed. Participants were only informed that they were to receive ‘one of two different tasks involving their smartphone’; i.e., were not told the condition (intervention/control) to which they were randomised. The researchers delivering the procedures were not blind to condition, as they needed to provide verbal instructions appropriate to the condition to which particiapnts were allocated.

Following randomisation, participants completed procedures according to condition assignment (see section “Treatment conditions”). Finally, participants were given an intrusive memory diary to assess the primary and secondary outcome (number of intrusions week 1 and week 5), and instructions for how to complete the diary. For further details regarding follow-ups at 1 week, 1, 3 and 6 months, see Supplementary Information and Table 1. Recruitment started in January 2019 and stopped in June 2019 after reaching the planned sample size. After 1 month, follow-up participants received a cinema voucher (value approximately USD 12) as reimbursement for their time. The last follow-up assessment was completed in December 2019.

### Ethical approval

The study was approved by the Regional Research Ethics Committee, Stockholm (EPN dnr: 2017/2215-31, amendments 2018/416-32, 2018/1435-32, 2018/2150-32 and 2019-01328). The study was pre-registered in a Clinical Trials Registry (CTR; clinical.trials.gov; number NCT03509792) on 26/04/2018. An independent clinical trials unit (Karolinska Trial Alliance) monitored the study to ensure compliance with Good Clinical Practice.

## Results

### Analytic approach

As an exploratory open-label pilot trial conducted to guide the design of future follow-up trials, we adopt a descriptive approach to reporting results. As planned, we conducted pilot intention-to-treat analyses to obtain an estimate of the between-group effect size of the intrusive memory diary measure, to guide power and sample size estimation for a full-scale follow-up RCT. Analyses were performed using R, Version 4.0.2^42^ (‘psych’ package, version 2.0.8, for descriptive analyses and ‘effsize’ package, version 0.5.1, for effect sizes) and power and sample size estimation was performed using STATA. De-identified summary data, codebook and R scripts are available on the Open Science Framework: osf.io/nma5q/.

Table 3 shows means, SDs and effect sizes (as Cohen’s *d* along with 95% CIs)^43^ of pre-specified outcome measures for the intervention and control groups at each assessment point for all outcome measures carried forward to a planned full RCT. Supplementary Tables 3 and 4 contain descriptive results pertaining to remaining measures (those not carried forward to a planned full RCT). See *Supplementary Information* for responses on Feedback Questionnaire and Implementation Feedback.

**Table 3:** Primary, secondary and other pre-specified outcome measures by condition.

Continuous outcome	Intervention (total *n* = 22)			Control (total *n* = 19)			Effect size	
	*n*	*Mean*	*SD*	*n*	*Mean*	*SD*	*d*	95% CI for *d*
*Primary outcome, Week 1*								
Number of intrusive memories (diary)	20	3.85^a^	8.57	19	7.37	7.88	0.43	−0.23, 1.08
*Secondary outcome, Week 5*								
Number of intrusive memories (diary)	18	0.28	0.57	18	2.89	6.43	0.57	−0.12, 1.26
*Secondary outcomes, 1 week*								
IES-R								
Intrusion subscale	16	4.12	4.13	17	8.53	6.23	0.83	0.09, 1.57
Avoidance subscale	16	2.38	2.42	17	8.06	7.85	0.97	0.22, 1.72
HADS								
Anxiety subscale	16	3.25	2.89	17	4.71	3.64	0.44	−0.28, 1.16
Depression subscale	16	1.62	1.89	17	3.06	3.36	0.52	−0.20, 1.24
Total	16	4.88	4.62	17	7.76	6.81	0.49	−0.23, 1.22
*Other outcomes, 1 week*								
SRHR	16	5.62	1.36	16	5.12	1.54	−0.34	−1.07, 0.38
SRSR	16	5.81	2.17	16	4.88	2.53	−0.40	−1.13, 0.33
Intrusion-related distress	13	2.00	2.55	16	3.38	3.42	0.45	−0.33, 1.22
Intrusion vividness	13	2.85	3.44	16	4.50	3.46	0.48	−0.30, 1.26
Concentration disruption	16	1.38	0.72	16	2.39	1.65	0.80	0.05, 1.55
*Secondary outcomes, 1 month*								
IES-R								
Intrusion subscale	16	1.88	2.39	18	3.78	3.72	0.60	−0.11, 1.32
Avoidance subscale	16	1.94	4.55	18	3.39	4.27	0.33	−0.38, 1.03
HADS								
Anxiety subscale	16	3.19	3.02	18	3.83	3.07	0.21	−0.49, 0.91
Depression subscale	16	2.12	3.24	18	2.72	3.63	0.17	−0.53, 0.87
Total	16	5.31	5.76	18	6.56	6.06	0.21	−0.49, 0.91
*Other outcomes, 1 month*								
SRHR	16	5.06	1.84	18	5.00	1.28	−0.04	−0.74, 0.66
SRSR	16	6.69	1.40	18	5.67	2.68	−0.47	−1.18, 0.24
Intrusion-related distress	13	1.69	3.45	16	1.25	2.24	−0.16	−0.92, 0.61
Intrusion vividness	13	1.08	2.29	16	1.88	2.53	0.33	−0.44, 1.10
Concentration disruption	15	1.27	1.03	18	1.83	1.50	0.43	−0.29, 1.15
*Secondary outcomes, 3 months*								
IES-R								
Intrusion subscale	15	1.33	2.13	18	2.33	2.83	0.39	−0.33,1.11
Avoidance subscale	15	0.80	1.26	18	1.61	2.85	0.36	−0.36,1.07
HADS								
Anxiety subscale	14	2.36	2.90	18	3.22	3.15	0.28	−0.45,1.02
Depression subscale	14	1.43	1.91	18	2.17	2.50	0.33	−0.41,1.06
Total	14	3.79	4.66	18	5.39	5.40	0.31	−0.42,1.05
*Other outcomes, 3 months*								
SRHR	14	5.57	1.34	18	5.44	0.86	−0.12	−0.84,0.61
SRSR	14	7.21	1.19	18	6.28	2.27	−0.50	−1.24,0.24
Concentration disruption	14	1.43	1.60	18	1.56	1.15	0.09	−0.64,0.82
*Secondary outcomes, 6 months*								
IES-R								
Intrusion subscale	12	1.33	3.47	16	0.75	1.13	−0.24	−1.03,0.55
Avoidance subscale	12	0.42	0.90	15	0.93	1.33	0.44	−0.36,1.25
HADS								
Anxiety subscale	12	2.08	2.50	16	3.44	3.22	0.46	−0.33,1.26
Depression subscale	12	1.08	1.68	15	1.87	1.81	0.45	−0.36,1.25
Total	12	3.17	4.02	15	5.53	4.66	0.54	−0.27,1.35
*Other outcomes, 6 months*								
SRHR	12	5.83	1.34	16	5.38	1.36	−0.34	−1.13,0.45
SRSR	12	7.25	0.87	16	5.12	2.66	−1.01	−1.85,−0.18
Concentration disruption	12	1.58	1.73	15	1.27	0.70	−0.25	−1.05,0.55

*CI* confidence interval, *HADS* Hospital Anxiety and Depression Scale, *IES-R* Impact of Event Scale–Revised, *SRHR* Self Rated Health Rating, *SRSR* Self Rated Sleep Rating.

^a^Table 3 reports the analysis including all participants (incl. outliers). Outliers in number of intrusive memories were inspected per group separately, and were identified based on standard criteria (i.e., >3 SD^51^). For week 1 data, one statistical outlier was detected within the intervention group (reported 37 intrusions). This participant did not receive correct protocol delivery in ED. For completeness, the outlier is included in the descriptive analyses reported here. No outliers were detected for week 5 data.

### Primary outcome

#### Number of intrusive memories of traumatic event (daily diary, week 1)

The primary outcome measure was the number of intrusive memories recorded in the daily diary in the week after receiving the intervention/control task. We obtained week 1 diaries from 39 (of 41) participants, i.e., an attrition rate of 4.9%. Participants in the intervention condition (*M* = 3.85, SD = 8.57, *n* = 20) recorded on average 3.52 fewer intrusive memories than those in the control condition (*M* = 7.37, SD = 7.88, *n* = 19), i.e., a 48% difference (*d* = 0.43, 95% CI: −0.23, 1.08, a small-medium effect size according to Cohen^43^).

**Fig. 2:**
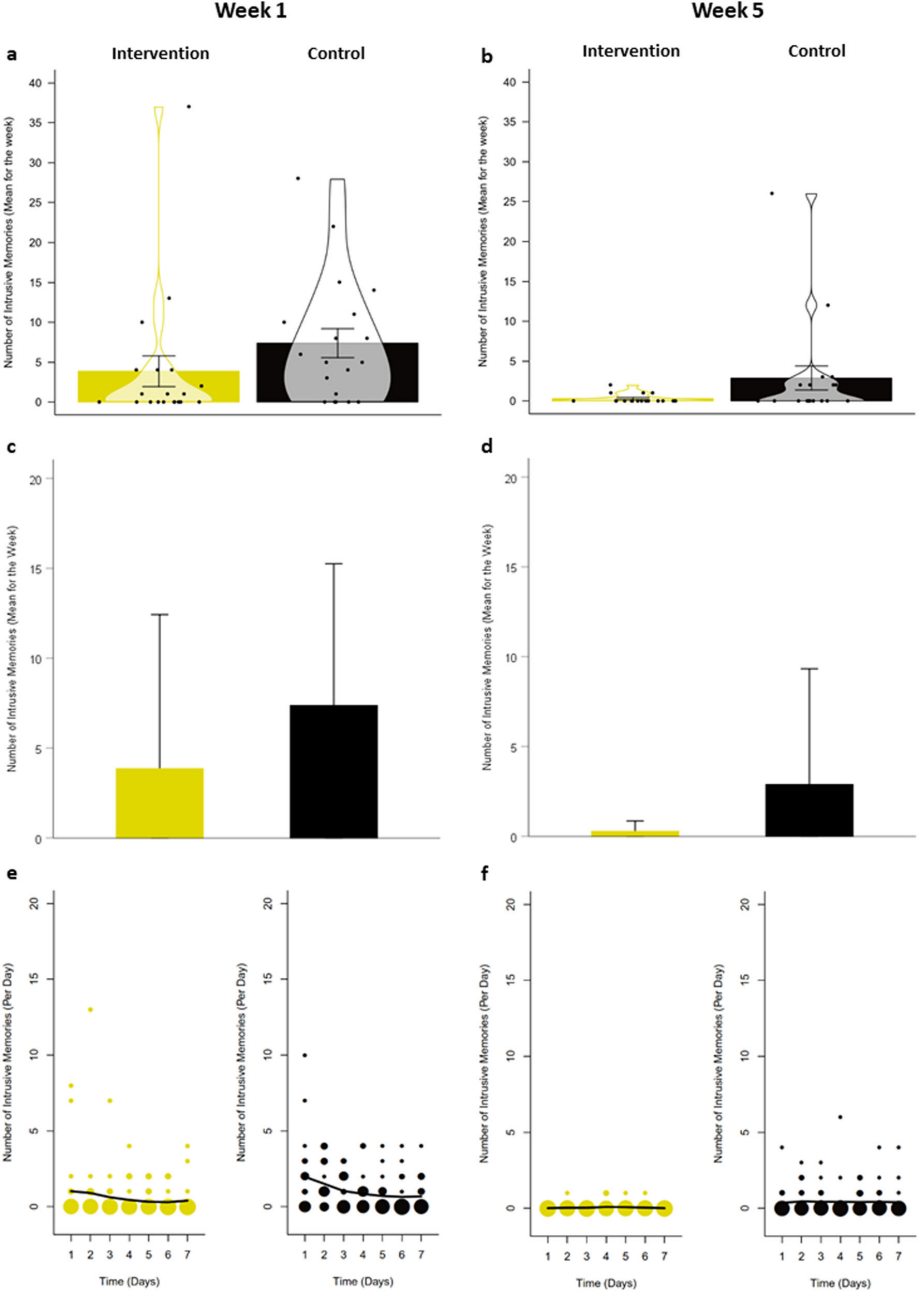
Number of intrusive memories of the traumatic event in the intervention and active control conditions. Intervention = cognitive task (trauma memory reminder cue plus Tetris computer game play using mental rotation); Active control condition = attention placebo task (listening to podcast). **a** Violin plots displaying mean number of intrusive memories recorded in a daily diary during week 1 after completing the intervention/control procedure following a traumatic event. Error bars depict standard errors. Points depict the total number of intrusive memories (per week) of each participant. Violins depict smoothed density. Plots were created with the pirate plot function of the ‘yarrr’ package (Version 0.1.5)^50^ in R^42^. **b** Violin plots displaying mean number of intrusive memories recorded in a daily diary during week 5 (as above). **c** Bar graphs displaying mean number of intrusive memories recorded in a daily diary during week 1 after completing the intervention/control procedure following a traumatic event. Error bars depict standard deviations (for comparison with Iyadurai et al.^3^, Fig. 2). **d** Bar graphs displaying mean number of intrusive memories recorded in a daily diary during week 5 (as above). **e** Frequency scattergraphs displaying the time course of the number of intrusive memories recorded in a diary during week 1 after completing the intervention/control procedure following a traumatic event. The size of the circles represents the number of participants who reported the indicated number of intrusive memories on that particular day, scaled separately for each condition (see also *Supplementary Information* for Fig. 2e). **f** Frequency scattergraphs displaying the time course of the number of intrusive memories recorded in a diary during week 5 (as above).

See Fig. 2a and c for visualisation of individual and summary level data on intrusive memories reported in the diary over week 1. To visually inspect the time course of intrusive memories, we plotted frequency scattergraphs showing their distribution on each day per condition (Fig. 2e).

### Secondary outcomes

#### Number of intrusive memories of traumatic event (daily diary, week 5)

As noted, we re-administered the diary after 1 month (a key difference to Iyadurai et al.^3^). We obtained week 5 diaries from 36 (of 41) participants, i.e., attrition rate of 12.2%. At week 5, participants in the intervention condition (*M* = 0.28, SD = 0.57, *n* = 18) reported on average 2.61 fewer intrusive memories than those in the control condition (*M* = 2.89, SD = 6.43, *n* = 18), i.e., a 90% difference (*d* = 0.57, 95% CI: −0.12, 1.26, a medium effect size, Fig. 2b, d, f).

#### Power analysis and sample size estimation for follow-up trials

Given the potential clinical value of examining longer-term effects of the intervention, we considered using the week 5 intrusive memory diary as the primary outcome in a follow-up trial. For power analysis and sample size estimation in such a follow-up trial, we can thus use the mean number of intrusive memories at week 5, i.e., mean intervention = 0.28 (*SD* = 0.57) versus mean control = 2.89 (*SD* = 6.43). Based on this two-group between group difference (*d* = 0.57, equivalent of ~0.5 standard deviation units), at power of 90% and alpha of 0.05, we would require a sample of size 65 participants per group (130 in total). Given the attrition rate of 12.2% here, a future trial could aim to recruit 146 participants in total. (There is a discrepancy of *n* = 2 between the *N* = 146 reported here and *N* = 148 in the CTR (NCT04185155) for the terminated follow-up RCT^44^, due to calculations being based on either *N* = 42 participants randomised or *N* = 41 final sample for analysis. *N* = 146 is based on the corrected attrition rate of 12.2% detected during the review process for this manuscript).

#### IES-R intrusion and avoidance subscales

Compared to control, participants in the intervention condition had lower scores on the IES-R intrusion subscale at 1 week, 1 and 3 months. At 6 months, scores on the IES-R intrusion subscale were lower in the control than the intervention condition. The intervention condition had lower scores on the IES-R avoidance subscale at all timepoints (1 week, 1, 3 and 6 months; Table 3).

#### HADS

Participants in the intervention condition had lower scores at all assessment timepoints than those in the control condition (Table 3).

### Other pre-specified outcome measures

#### Credibility/expectancy questionnaire

On day 1, ratings of credibility (i.e., whether participants expected the intervention/control task to work) were similar and in the mid-range across conditions (intervention: *M* = 25.48, SD = 15.04, *n* = 21; control: *M* = 26.37, SD = 9.82, *n* = 19, *d* = 0.07, 95% CI: −0.57, 0.71).

#### Self Rated Health ratings (SRHR)

Participants in the intervention condition had higher SRHR scores at all timepoints than those in the control condition (Table 3).

#### Self Rated Sleep ratings (SRSR)

Participants in the intervention condition had higher SRSR scores at all timepoints than those in the control condition (Table 3).

#### Adverse events

No adverse events or potential side effects that could be related to study procedures were reported. No serious adverse events were reported.

#### Characteristics of intrusive trauma memories

At 1 week, intrusions were rated as less distressing and vivid, and causing less concentration disruption in the intervention condition. At 1 month, those in the intervention condition who experienced intrusions (*n* = 4/18) rated them as more distressing and less vivid than controls with intrusions (*n* = 8/18). Later follow-ups only included concentration disruption ratings, with lower scores in the intervention condition at 3 months and the opposite (i.e. higher ratings of disruption) at 6 months (effect sizes small to very small; Table 3).

### Other outcomes

#### Treatment adherence and attrition

Compliance to the assigned task condition was high in both conditions. In the intervention condition, one participant did not complete the memory reminder cue and two did not play Tetris. All participants in the control condition listened to the podcast. Self-reported accuracy ratings for diary completion were high in both conditions (week 1: intervention: *M* = 8.67, SD = 1.54, *n* = 15; control: *M* = 8.22, SD = 1.60, *n* = 16; week 5: intervention: *M* = 8.21, SD = 2.12, *n* = 14; control: *M* = 8.56, SD = 1.67, *n* = 16).

Attrition (*N* = 41) was 4.9% for the primary outcome measure (week 1 diary). For secondary/other outcomes, attrition was 19.5% at 1 week, 17.1% at 1 month (12.2% for the week 5 diary), 19.5% at 3 months and 31.7% at 6 months.

## Discussion/conclusion

This exploratory open-label pilot RCT investigated the feasibility and effects of a brief cognitive task intervention (including Tetris gameplay as one of several components) on intrusive memories of trauma in individuals who presented to the ED. Relative to the active control condition, trauma-exposed individuals who received the intervention reported fewer intrusive memories in the subsequent week (48% fewer in intervention compared to control condition), consistent with the direction of previous findings with analogue^22^ and real-world^3^ trauma. A between-group difference was maintained 1 month later; i.e., for intrusions reported in the week 5 diary (90% fewer in intervention compared to control condition). Notably, ratings of credibility (i.e., whether participants expected the intervention/control task to work) were similar and in the mid-range in both conditions, suggesting expectancy effects were unlikely to drive group differences. Results establish the daily diary as a feasible tool with which to monitor intrusions not only at week 1 but also at week 5 post-intervention.

The overall pattern of means suggests that participants who received the intervention (compared to control) reported less intrusion-related distress (IES-R) and some possible functional improvements (e.g., concentration). As a brief (single session) intervention, initial indication of longer-term effects than previous studies (i.e., beyond 1 week) on aspects of psychological functioning warrant further exploration. That said, we note the reverse pattern for intrusion-related distress at 1 month, and IES-R and concentration disruption at 6 months. Overall, we interpret findings with caution given the exploratory nature of the study, the relatively small sample, and attrition over time.

Participants responded positively to taking part and rated both conditions overall as not burdensome and quite easy to complete. None provided negative feedback in open-ended comments or follow-up telephone interviews (*Supplementary Information*). Most patients approached welcomed the opportunity to participate in the study during waiting-time in the ED, with 88% (101 of 115 eligible participants) willing to complete study procedures. International clinical guidelines for PTSD^10^ report treatment acceptability using rates of treatment discontinuation. Here, 20 of 22 participants (91%) randomised to the intervention condition completed the treatment.

Feedback from ED staff indicated they were receptive to the study; they supported identification of eligible patients, and appreciated how study procedures fitted around patients’ medical care. In line with our earlier work exploring feasibility of recruiting in the ED^33^, staff welcomed that participants underwent study procedures during the naturally occurring (often long) waiting-times in the ED, as it kept them actively engaged. Overall, this suggests feasibility of delivery in a different international context (Sweden) to that in which the intervention was developed (UK), again in a hospital ED/waiting areas and outside a traditional psychotherapy setting.

Intrusive memories were reported by participants as a result of a broad range of traumas, from minor fall accidents to severe injuries and physical assaults. This speaks to the relevance of a broad recruitment approach, and suggests successful implementation in a mixed trauma sample. One next step will be to investigate whether the intervention can be delivered remotely (e.g., digitally rather than face-to-face), which will have utility when in person meetings are not possible and will maximise the scope of the intervention to prevent intrusive memories at scale.

The unprecedented circumstances arising from the COVID-19 pandemic emphasise the need for remote delivery^45^. In light of the favourable outcomes of this pilot, we had commenced the abovementioned planned follow-up RCT in the same ED. However, data collection was swiftly suspended in light of the pandemic due to infection risks of COVID-19, and the trial was terminated (CTR: NCT04185155)^44^. In response to informal enquiries from ED staff about using the intervention to address their *own* intrusive memories of trauma, during the pandemic we developed a new study to evaluate the impact of the intervention for frontline healthcare workers (CTR: NCT04460014)^45–47^ e.g., for intrusive memories as a result of work with critically ill/dying COVID-19 patients in intensive care. This follow-up RCT builds on this pilot trial and the intervention procedures piloted here, however it uses remote delivery (i.e., digitally rather than in person, using instructional videos^48^ and remote researcher support) due to COVID-19 infection risk. The design and sample size estimation are guided by the between-group effect size in the current pilot findings. Going forward, we look to possibilities to evaluate the intervention in further follow-up trials, in which careful consideration of implementation and adaption to specific context/populations will be important.

Procedures for training the research team to deliver the intervention and monitoring outcomes worked well, and real-time supervision (via phone from the university to the ED) was successfully implemented. Feedback from students indicated the importance of role-play as part of training, and that the imagery-based metaphor ‘acting like your favourite nurse’ helped them deliver the intervention in a swift and practical manner. They noted that it was important to establish a relationship with ED staff on-site to facilitate recruitment and understanding of the ED context, and that instructional videos and further development of the brief checklist for delivering the intervention would be helpful as part of future training. Thus, our training procedures for research staff require more formalisation, write-up and development so that they can be more readily shared and used to train new staff members. As the intervention is in its relative infancy, we consider the development of formalised training and fidelity procedures to be a critical next step in translational research, to ensure effective and consistent delivery of the intervention and study procedures (e.g., daily diary) in subsequent research trials and to facilitate implementation. Adequate training is vital for adaptation to specific contexts, implementation across different settings/centres, and to aid reproducibility efforts^49^.

Compared to traditional psychotherapy, costs of the intervention in its current form are low, as it involves one main session (rather than multiple sessions), and does not rely on delivery by a qualified clinician. We are exploring possibilities such as digital training procedures (to facilitate delivery), which could further reduce costs. Furthermore, the only technical device needed to complete the intervention is the participant’s own smartphone. Future trials could include health economics evaluations to formally establish the costs of the intervention.

We note various limitations of this exploratory open-label pilot RCT in addition to those previously mentioned. First, whilst conceptual accounts of memory (re)consolidation inspired aspects of the behavioural intervention, this study cannot elucidate mechanisms. Second, we opted not to report findings on a measure of sensory features of intrusive memories as items were poorly understood by participants. We stratified participants based on their ratings of perceived threat during the traumatic event, and had intended to do so in a follow-up RCT. However, our pilot data provided no indication of utility of this approach (see Supplementary Fig. 1). Finally, rates of attrition for secondary outcomes (particularly at 6 months) compromise our capacity to make meaningful between-condition comparisons at longer timepoints.

In sum, the global prevalence of trauma necessitates preventive approaches that are simple to deliver at scale. This one-session behavioural intervention resulted in fewer intrusive trauma memories at 1 week and 1 month post-trauma for individuals who presented at the ED. Our next step will be to investigate whether the intervention can contribute to the mental health of frontline healthcare staff by preventing the persistence of intrusive memories of trauma they experience in the context of the pandemic.

## Supplementary information

Supplementary materials

Supplementary Figure 1
