# Supplementary material for: Reducing intrusive memories after trauma via a brief cognitive task intervention in the hospital emergency department: an exploratory pilot randomised controlled trial
Source: Transl Psychiatry. 2021 Jan 11;11:30. doi: 10.1038/s41398-020-01124-6 (PMC7798383; doi:10.1038/s41398-020-01124-6)
Supplement: Supplementary file 1 — Supplementary materials [file 41398_2020_1124_MOESM1_ESM.docx]

## **Supplementary Information**

Reducing Intrusive Memories After Trauma via a Brief Cognitive Task Intervention in the Hospital Emergency Department:

an Exploratory Pilot Randomised Controlled Trial

Marie Kanstrup^*^, Laura Singh^*^, Katarina E. Göransson, Julia Widoff, Rod S. Taylor, Beau Gamble, Lalitha Iyadurai, Michelle L. Moulds & Emily A. Holmes

* co-shared first authorship

De-identified summary data, codebook and R scripts are available on the Open Science Framework: osf.io/nma5q/.

**Supplementary Materials and Methods**

**Measures and Materials**

***Information about the participant and the traumatic event.***

***Traumatic event details.*** Information about the traumatic event associated with admission to the ED and participants’ treatment in the ED was obtained from participants and in collaboration with ED staff.

***Demographic information.*** This was collected from participants; included gender, occupation, civil status, education, yearly income, and country of origin for the participant and for their parents.

***Mental health/medical/trauma history.*** Participants were briefly asked about previous trauma, any current and/or past mental and medical problems, and family psychiatric history.

***Ratings of perceived threat*** ***to self/other.*** Participants rated the following items: *‘to what extent did you feel your life was in danger?’, ‘to what extent did you feel that you were at risk for serious injury?’, ‘to what extent did you feel that someone else’s life was in danger?’, ‘to what extent did you feel that someone else was at risk of serious injury?’* on a 0-10 scale, where 0 = not at all, 10 = extremely (see Procedural changes section below).

***Peritraumatic Dissociative Experiences Questionnaire (PDEQ)***. To assess dissociative symptoms during the trauma. The 10 items are rated from 1 = not at all true, to 5 = completely true^1^.

***Peritraumatic Distress Inventory (PDI)***. To assess emotional responses related to the trauma. The 13 items are rated from 0 = not at all, to 4 = completely^2^.

***Injury Severity.*** Ratings of Injury Severity were obtained retrospectively in collaboration with ED staff, using a template based on the Injury Severity Score online version^3^. Agreement for separate injury codings between raters (JW and a nurse) was 80.5%, and increased to 100% after discussion. Inconsistencies were primarily in the lower range of the scale, concerning whether an injury was to be assessed as ‘no injury’, or ‘minor injury’.

***Secondary Outcome Measures.***

***Perceived Stress Scale (PSS).*** To assess perception of stress and stressful appraisal of life situations. The 14-items are rated from 0 = never, to 4 = very often^4^.

***Work and Social Adjustment Scale (WSAS).*** This 5-item measure, anchored to the traumatic event, assessed associated functional impairment (e.g., in social, work, leisure domains). Lower scores indicate less impairment^5^.

***Other Pre-specified Outcome Measures***

***Characteristics of intrusive trauma memories.*** Intrusion vividness (i.e., ‘*How vivid were your intrusive memories?*’) and distress (i.e., ‘*How distressing were your intrusive memories?*’) (both on a scale where 0 = not at all, 10 = extremely) were rated on the final day of each diary monitoring week (i.e., weeks 1 and 5). Concentration disruption (‘*During the past week, how much did intrusive memories disrupt your concentration?*’) was rated on a scale from 1 (not disruptive at all) to 9 (very disruptive) in the electronic online platform at week 1, week 5, 3- and 6-months, within the Feedback Questionnaire about Participation (reported in more detail below).

***Feedback Questionnaire about Participation*.** This included 22 bespoke items to obtain additional information relevant to the current study. Items were slightly modified over the course of the study, and included questions about the impact of intrusions on for example concentration, whether participants had received other psychological/medical treatments since being contacted in the ED, and level of social support.

Participants were also asked about their experience of taking part in the study (e.g., whether they appreciated having something to do while waiting in the ED), and to rate how easy or burdensome they found completing the task in the ED (1=not at all easy/burdensome, 5=quite easy/burdensome, 9=very easy/burdensome). They were also asked to provide any other comments.

***Implementation feedback.*** Observation and qualitative interviews with participants, study team and staff regarding implementation of the procedures.

***Sensory modalities of intrusive trauma memories.*** 7 items measuring sensory modality of intrusive memories.^6^

***Mini International Neuropsychiatric Interview***. MINI, Version 7.00, Section H^7^. A 19-item structured interview administered via telephone at 1-, 3-, and 6-months to assess current PTSD symptoms.

**Procedure**

At baseline, participants additionally provided details about psychiatric/trauma history and ratings of perceived threat to self/other, and baseline self-report measures.

Participants were contacted for follow-up assessments at one week, 1-, 3-, and 6-months post-trauma. Follow-ups consisted of telephone calls and questionnaires delivered via an electronic online platform (see Table 2 for an overview of questionnaires administered at each assessment point). For participants who preferred pen-and-paper questionnaires, these were sent by post. At each assessment, participants completed a feedback questionnaire asking about the experience of participating in the study, and were asked about any side effects or adverse events via telephone calls. Ratings of injury severity (ISS) were obtained in collaboration with ED staff.

Participants were sent a standard letter after the final follow-up (6-months) which included links and telephone numbers for information about persisting mental health symptoms after trauma (e.g., PTSD) and evidence-based treatments.

**Supplementary Results**

**Other Pre-specified Outcome Measures.**

***Feedback Questionnaire about Participation.*** Ratings on the item *How easy did you find it to do the task in the emergency department?* were similar for the two conditions at each timepoint, and increased comparably as the study progressed (Supplementary Table 5). In both conditions, ratings of the extent to which the task was burdensome were low (*How burdensome did you find doing the task in the emergency department?*; Supplementary Table 5).

Participants in both conditions indicated high levels of social support (Supplementary Table 5). Participants in the two conditions reported comparable receipt of other treatment following the trauma. Five participants in the intervention condition reported receiving other treatment (analgesic medicine, *n*=4; doctor and psychotherapist, *n*=1), while three participants in the control condition received other treatment (all took analgesic medicine).

When asked if they had any additional comments, no participants provided negative comments about study participation. Positive comments included that the intervention was a helpful distraction *“I disconnected from all other thoughts and that was good”* (intervention, 6-month follow up). Feedback also suggested the possibility of reducing the number of outcome measures *“It is a lot of questions and several are a bit fuzzy”* (intervention, 6-month follow-up). One participant mentioned that their injury/pain made participation difficult (intervention, 6-month follow-up).

***Implementation feedback.*** No participant expressed negative comments about study participation in telephone interviews. Feedback included that participants considered it *“beneficial to take part”* (control, 6-month follow-up) and *“not burdensome at all”* (control, 6-month follow-up). Further, study participation was described as very good in the sense that *“hospital staff don’t have time to talk with you, it is good to talk so you don’t sit and feel sorry for yourself”* (control, 6-month follow-up). One participant explicitly thanked the study team for having had the opportunity to take part: *“it is not until now that I understand how good this was, I needed the distraction”* [*when at the ED*] (intervention, 6-month follow-up).

Implementation of study procedures worked well, in line with prior feasibility work^8^ . Staff were keen to support recruitment and help research staff to identify eligible patients. The expansion of study sites to include the nearby walk-in centre as well as the main ED was a result of suggestions from ED staff. Presentations about the project were well-attended by staff, and at study end, ED staff presented the project at a healthcare conference. The MSc students who assisted with data collection and delivered the intervention provided feedback on training procedures; e.g., regarding key learning points for how to deliver key components of the intervention.

**Figure 2e.**

Note that one participant in the intervention condition only completed day 1-4 of the week 1 diary and is not included in this frequency scattergraph (Figure 2e) due to not being able to visualize incomplete data with the R Script. They are included in Figures 2a and c. Their numbers of intrusive memories were day 1=0, day 2=0, day 3=0, day 4=1.

**Procedural Changes During Piloting**

Four modifications were made to the procedure as part of piloting during the course of the study. First, in the original protocol participants were seen in the ED within 6 hours of the trauma. Based on recent experimental findings^9^, and to increase the recruitment rate, on 8/5/2019 (following the recruitment of 18 participants) we tested extending this criterion so that participants could receive the intervention/control task within 72 hours of the trauma (yielding 11 additional participants).

Second, on 17/6/19, after having recruited 32 participants, of whom 7 presented at the ED >6 hours after trauma, a change was made to the intervention procedure. After this date, participants who presented at the ED >6 hours after trauma and were randomised to the intervention condition (n=2), were asked to undertake a minimum Tetris gameplay of at least one uninterrupted period of 10 min and for ~20 min for *each ‘hotspot’* *separately*, resulting in a longer play duration to adjust for treating individual hotspots in the post-consolidation phase^10^.

Third, the first three participants in the control group were given a list of podcasts on a range of topics, and given the option of choosing which one they listened to. On 4/1/19 a change was made to the control procedure such that all further participants listened to just one pre-selected podcast series (i.e., a specific episode on Swedish philosophy, ‘Filosofiska rummet – Vägen till svensk filosofi’^11^). This change was made in order to minimise the likelihood of participants being exposed to content provoking mental visual images apparent in other podcasts if there was a wide range to select from.

Fourth, ratings about perceived threat to self/other were modified. After 7 participants were recruited, we expanded the initial questions of threat to life for self/others (used in^12^) to include threat of serious injury to self/others (for the next 13 participants). For the remaining 21 participants, these questions were separated into four questions for clarification (life threat to self, life threat to others, threat of serious injury to self, and threat of serious injury to others).

In summary, modifications to the protocol made during the course of the study proved challenging for research assistants because different protocols for the intervention procedure had to be used depending on whether hotspots needed to be targeted separately. Accordingly, the (now terminated) RCT commenced in the ED used the initial 6-hour criterion to keep study procedures the same for all participants.

**Supplementary Tables**

**Suppl. Table 1.** Reasons patients were screened out before being approached (n=309).

| **Reason patient was screened out before being approached** | **n** |
| --- | --- |
| Had been in ED > 6h^[[1]](#footnote-2)^ | 95 |
| No event^[[2]](#footnote-3)^ | 75 |
| Not able to complete study procedures (e.g. patient/researcher unavailable)^[[3]](#footnote-4)^ | 43 |
| *SUBTOTAL* | *213* |
| Other^[[4]](#footnote-5)^ |  |
| Aged under 18 | 3 |
| Event did not meet the DSM-5 criterion A for PTSD | 0 |
| No memory of accident | 7 |
| Not fluent in Swedish | 18 |
| Not alert and oriented | 7 |
| Insufficient physical mobility to use intervention platform | 4 |
| No access to an internet enabled smartphone | 1 |
| Could not be seen in the ED within 72 hours after the traumatic event^[[5]](#footnote-6)^ | 16 |
| Loss of consciousness of > 5 minutes | 7 |
| Current intoxication | 6 |
| Reported history of severe mental illness | 3 |
| Current substance abuse or neurological condition | 23 |
| Currently suicidal | 1 |
| *SUBTOTAL OTHER* | *96* |
| *TOTAL* | *309* |

Abbreviations: DSM-5 = Diagnostic and Statistical Manual of Mental Disorders, 5^th^ Edition.

**Suppl. Table 2.** Reasons patients – all of whom experienced an event resulting in admission to the emergency department – were not willing to complete study procedures (n=14) or were excluded during eligibility assessment step 1 (n=54) and eligibility assessment step 2 (n=5) after being approached (Total n=73).

| **Reason patient was excluded during eligibility assessment after being approached^[[6]](#footnote-7)^** | **Excluded during eligibility assessment step 1**  **(n=68)** | **Excluded during eligibility assessment step 2**  **(n=5)** |
| --- | --- | --- |
| Aged under 18 | 0 | 0 |
| No event | 0 | 0 |
| Event did not meet the DSM-5 criterion A for PTSD | 33 | 0 |
| Could not be seen in the ED within 6 hours of the traumatic event | 0 | 1 |
| No memory of accident | 1 | 0 |
| Not fluent in Swedish | 4 | 0 |
| Not alert and oriented | 1 | 0 |
| Insufficient physical mobility to use intervention platform | 0 | 0 |
| Not willing to complete study procedure | 14^[[7]](#footnote-8)^ | 0 |
| Not able to complete study procedures | 8 | 2 |
| No access to an internet enabled smartphone | 1 | 0 |
| Could not be seen in the ED within 72 hours of the traumatic event | 6 | 0 |
| Loss of consciousness of > 5 minutes | 0 | 0 |
| Current intoxication | 0 | 0 |
| Reported history of severe mental illness | 0 | 2 |
| Current substance abuse or neurological condition | 0 | 0 |
| Currently suicidal | 0 | 0 |
| TOTAL | 73 | |

Abbreviations: DSM-5 = Diagnostic and Statistical Manual of Mental Disorders, 5^th^ Edition.

**Suppl. Table 3**. Primary, Secondary and other Pre-specified Outcome Measures, which were not included in the planned (now terminated) full RCT

| *Continuous outcome* |  | *Intervention (total n = 22)* | |  | *Control (total n = 19)* | |
| --- | --- | --- | --- | --- | --- | --- |
|  | *n* | *Mean* | *SD* | *n* | *Mean* | *SD* |
|  |  |  |  |  |  |  |
| *Secondary outcomes, one week* |  |  | |  |  | |
| IES-R |  |  |  |  |  |  |
| Hyperarousal subscale | 16 | 3.00 | 2.73 | 17 | 4.41 | 5.00 |
| Total | 16 | 9.50 | 7.90 | 17 | 21.00 | 17.04 |
| WSAS | 16 | 17.06 | 11.26 | 16 | 14.12 | 10.67 |
|  |  |  |  |  |  |  |
| *Secondary outcomes, 1-month* |  |  |  |  |  |  |
| IES-R |  |  |  |  |  |  |
| Hyperarousal subscale | 16 | 1.38 | 2.53 | 18 | 2.22 | 3.23 |
| Total | 16 | 5.19 | 8.77 | 18 | 9.39 | 9.70 |
| WSAS | 16 | 8.62 | 10.53 | 18 | 9.56 | 12.64 |
| PSS | 16 | 19.94 | 9.07 | 18 | 20.72 | 10.79 |
|  |  |  |  |  |  |  |
| *Other outcomes, 1-month* |  |  |  |  |  |  |
| MINI 7.0.0 – Section H^[[8]](#footnote-9)^ | 14 | 2.36 | 2.21 | 17 | 2.41 | 2.06 |
|  |  |  |  |  |  |  |
| *Secondary outcomes, 3-months* |  |  |  |  |  |  |
| IES-R |  |  |  |  |  |  |
| Hyperarousal subscale | 15 | 1.07 | 2.22 | 18 | 0.94 | 1.59 |
| Total | 15 | 3.20 | 4.95 | 18 | 4.89 | 6.80 |
| WSAS | 14 | 5.50 | 8.92 | 18 | 4.72 | 6.39 |
|  |  |  |  |  |  |  |
| *Other outcomes, 3-months* |  |  |  |  |  |  |
| MINI 7.0.0 – Section H | 15 | 1.20 | 1.57 | 14 | 2.07 | 2.73 |
|  |  |  |  |  |  |  |
| *Secondary outcomes, 6-months* |  |  |  |  |  |  |
| IES-R |  |  |  |  |  |  |
| Hyperarousal subscale | 12 | 0.67 | 2.02 | 16 | 0.62 | 1.78 |
| Total | 12 | 2.42 | 6.29 | 15 | 2.20 | 3.17 |
| WSAS | 12 | 4.00 | 9.22 | 16 | 4.69 | 8.85 |
|  |  |  |  |  |  |  |
| *Other outcomes, 6-months* |  |  |  |  |  |  |
| MINI 7.0.0 – Section H | 15 | 1.07 | 1.94 | 15 | 1.47 | 1.81 |
|  |  |  |  |  |  |  |
| *Categorical outcomes* | *n* | *%* |  | *n* | *%* |  |
|  |  |  |  |  |  |  |
| *PTSD MINI 7.0.0 – Section H* |  |  |  |  |  |  |
| 1-month | 0/15 | 0 |  | 0/17 | 0 |  |
| 3-months | 0/16 | 0 |  | 0/17 | 0 |  |
| 6-months | 0/15 | 0 |  | 0/15 | 0 |  |

*Note.* IES-R, Impact of Event Scale – Revised^13^; MINI, Mini-International Neuropsychiatric Interview^7^; PSS, Perceived Stress Scale^4^; WSAS, Work and Social Adjustment Scale^5^.

**Suppl. Table 4**. Participant feedback on participation

| *Continuous outcome* |  | *Intervention (total n = 22)* | |  | *Control (total n = 19)* | |
| --- | --- | --- | --- | --- | --- | --- |
|  | *n* | *Median* | *Range* | *n* | *Median* | *Range* |
|  |  |  |  |  |  |  |
| *Feedback on acceptability, 1-month* |  |  |  |  |  |  |
| “How easy did you find it to do the task in the emergency department?” 1 (not at all easy) 5 (quite easy) 9 (very easy) | 15 | 5 | 3 to 9 | 13 | 5 | 2 to 9 |
| “How burdensome did you find doing the task in the emergency department?” 1 (not at all burdensome) 5 (quite burdensome) 9 (very burdensome) | 15 | 1 | 1 to 5 | 13 | 2 | 1 to 7 |
| “How easy did you find doing the intervention on your own smartphone after the traumatic event?” 1 (not at all easy) 5 (quite easy) 9 (very easy) | 15 | 6 | 2 to 9 | 18 | 6.5 | 2 to 9 |
| “How helpful did you find doing the intervention after the traumatic event?” 1 (not at all helpful) 5 (quite helpful) 9 (very helpful) | 15 | 5 | 1 to 9 | 13 | 4 | 1 to 9 |
| ”If you experienced another traumatic event in the future, how willing would you be doing the intervention if it was offered as a way to prevent intrusive memories?” 1 (not at all willing) 5 (quite willing) 9 (very willing) | 15 | 5 | 1 to 9 | 13 | 6 | 1 to 9 |
| “How easy did you find participating in the study?” 1 (not at all easy) 5 (quite easy) 9 (very easy) | 15 | 5 | 1 to 9 | 18 | 5 | 3 to 9 |
| “How useful do you believe your relatives thought it was for you to participate in the study?” 1 (not at all useful) 5 (quite useful) 9 (very useful) | 12 | 2 | 1 to 9 | 13 | 2 | 1 to 9 |
|  |  |  |  |  |  |  |
| *Feedback on acceptability, 3-months* |  |  |  |  |  |  |
| “How easy did you find it to do the task in the emergency department?” 1 (not at all easy) 5 (quite easy) 9 (very easy) | 14 | 9 | 1 to 9 | 18 | 6 | 2 to 9 |
| “How burdensome did you find doing the task in the emergency department?” 1 (not at all burdensome) 5 (quite burdensome) 9 (very burdensome) | 14 | 1 | 1 to 4 | 18 | 2 | 1 to 9 |
| “How easy did you find doing the intervention on your own smartphone after the traumatic event?” 1 (not at all easy) 5 (quite easy) 9 (very easy) | 14 | 9 | 5 to 9 | 18 | 8 | 1 to 9 |
| “How helpful did you find doing the intervention after the traumatic event?” 1 (not at all helpful) 5 (quite helpful) 9 (very helpful) | 14 | 2.5 | 1 to 5 | 18 | 5 | 1 to 9 |
| ”If you experienced another traumatic event in the future, how willing would you be doing the intervention if it was offered as a way to prevent intrusive memories?” 1 (not at all willing) 5 (quite willing) 9 (very willing) | 14 | 5 | 1 to 9 | 18 | 6 | 1 to 9 |
| “How easy did you find participating in the study?” 1 (not at all easy) 5 (quite easy) 9 (very easy) | 14 | 5.5 | 1 to 9 | 18 | 6.5 | 1 to 9 |
| “How useful do you believe your relatives thought it was for you to participate in the study?” 1 (not at all useful) 5 (quite useful) 9 (very useful) | 14 | 1.5 | 1 to 9 | 18 | 5 | 1 to 9 |
|  |  |  |  |  |  |  |
| *Feedback on acceptability, 6-months* |  |  |  |  |  |  |
| “How easy did you find it to do the task in the emergency department?” 1 (not at all easy) 5 (quite easy) 9 (very easy) | 12 | 9 | 3 to 9 | 15 | 8 | 3 to 9 |
| “How burdensome did you find doing the task in the emergency department?” 1 (not at all burdensome) 5 (quite burdensome) 9 (very burdensome) | 12 | 1.5 | 1 to 4 | 15 | 2 | 1 to 7 |
| “How easy did you find doing the intervention on your own smartphone after the traumatic event?” 1 (not at all easy) 5 (quite easy) 9 (very easy) | 12 | 9 | 3 to 9 | 15 | 8 | 4 to 9 |
| “How helpful did you find doing the intervention after the traumatic event?” 1 (not at all helpful) 5 (quite helpful) 9 (very helpful) | 12 | 3.5 | 1 to 6 | 15 | 5 | 1 to 9 |
| ”If you experienced another traumatic event in the future, how willing would you be doing the intervention if it was offered as a way to prevent intrusive memories?” 1 (not at all willing) 5 (quite willing) 9 (very willing) | 12 | 5.5 | 1 to 9 | 14 | 5.5 | 1 to 9 |
| “How easy did you find participating in the study?” 1 (not at all easy) 5 (quite easy) 9 (very easy) | 12 | 6.5 | 1 to 9 | 15 | 8 | 1 to 9 |
| “How useful do you believe your relatives thought it was for you to participate in the study?” 1 (not at all useful) 5 (quite useful) 9 (very useful) | 12 | 1.5 | 1 to 7 | 15 | 3 | 1 to 9 |
|  |  |  |  |  |  |  |
| *Feedback on treatment credibility, 1-month* |  |  |  |  |  |  |
| “To what degree do you believe the intervention increased or decreased your intrusive memories of the event?” -9 (extreme decrease) 0 (no effect) 9 (extreme increase) | 15 | 0 | -6 to 0 | 18 | 0 | -9 to 3 |
| “How do you believe the intervention affected your mood?” -4 (for the worse) 0 (not at all) 4 (for the better) | 15 | 0 | 0 to 4 | 18 | 0 | -3 to 4 |
|  |  |  |  |  |  |  |
| *Feedback on treatment credibility, 3-months* |  |  |  |  |  |  |
| “To what degree do you believe the intervention increased or decreased your intrusive memories of the event?” -9 (extreme decrease) 0 (no effect) 9 (extreme increase) | 14 | 0 | -5 to 4 | 18 | 0 | -9 to 6 |
| “How do you believe the intervention affected your mood?” -4 (for the worse) 0 (not at all) 4 (for the better) | 14 | 0 | 0 to 3 | 18 | 0 | 0 to 4 |
|  |  |  |  |  |  |  |
| *Feedback on treatment credibility, 6-months* |  |  |  |  |  |  |
| “To what degree do you believe the intervention increased or decreased your intrusive memories of the event?” -9 (extreme decrease) 0 (no effect) 9 (extreme increase) | 12 | 0 | -9 to 0 | 15 | 0 | -9 to 5 |
| “How do you believe the intervention affected your mood?” -4 (for the worse) 0 (not at all) 4 (for the better) | 12 | 0 | 0 to 2 | 15 | 0 | 0 to 4 |
|  |  |  |  |  |  |  |
| *Feedback on social support, 1-month* |  |  |  |  |  |  |
| “How much support have you received from family and friends after the traumatic event?” 1 (no support) 5 (quite some support) 9 (much support) | 15 | 9 | 5 to 9 | 18 | 8.5 | 2 to 9 |
|  |  |  |  |  |  |  |
| *Feedback on social support, 3-months* |  |  |  |  |  |  |
| “How much support have you received from family and friends after the traumatic event?” 1 (no support) 5 (quite some support) 9 (much support) | 14 | 9 | 1 to 9 | 18 | 6 | 1 to 9 |
|  |  |  |  |  |  |  |
| *Feedback on social support, 6-months* |  |  |  |  |  |  |
| “How much support have you received from family and friends after the traumatic event?” 1 (no support) 5 (quite some support) 9 (much support) | 12 | 9 | 1 to 9 | 15 | 9 | 2 to 9 |
|  |  |  |  |  |  |  |
| *Feedback on intervention use at home, one week* |  |  |  |  |  |  |
| “How often did you do the intervention after experiencing intrusive memories from the traumatic event?” 1 (never) 5 (half the times) 9 (always) | 16 | 1 | 1 to 8 | 8 | 1 | 1 to 3 |
|  |  |  |  |  |  |  |
|  | *Total n* | *n* | *%* | *Total n* | *n* | *%* |
|  |  |  |  |  |  |  |
| *Other feedback, one week* |  |  |  |  |  |  |
| “Received other treatment due to the traumatic event” | 16 | 5 | 31.3 | 16 | 3 | 18.8 |
|  |  |  |  |  |  |  |
| *Other feedback, 1-month* |  |  |  |  |  |  |
| ”Have you played any other visuospatial game that is similar to Tetris since the traumatic event” | 15 | 2 | 13.3 | 18 | 2 | 11.1 |
|  |  |  |  |  |  |  |
| *Other feedback, 3-months* |  |  |  |  |  |  |
| ”Have you played any other visuospatial game that is similar to Tetris since the traumatic event” | 14 | 0 | 0.0 | 18 | 1 | 5.6 |
|  |  |  |  |  |  |  |
| *Other feedback, 6-months* |  |  |  |  |  |  |
| ”Have you played any other visuospatial game that is similar to Tetris since the traumatic event” | 12 | 2 | 16.7 | 15 | 3 | 20.0 |

**Supplementary References**

1. Birmes, P., Brunet, A., Carreras, D., Ducassé, J.L., Charlet, J.P., Lauque, D., et al., The predictive power of peritraumatic dissociation and acute stress symptoms for posttraumatic stress symptoms: a three-month prospective study*.* *Am. J. Psychiatry*. **160**(7), 1337-1339 (2003).

2. Brunet, A., Weiss, D.S., Metzler, T.J., Best, S.R., Neylan, T.C., Rogers, C., et al., The Peritraumatic Distress Inventory: A Proposed Measure of PTSD Criterion A2*.* *Am. J. Psychiatry*. **158**, 1480-1485 (2001).

3. Baker, S.P., O'Neill, B., Haddon, W., Jr., and Long, W.B., The injury severity score: a method for describing patients with multiple injuries and evaluating emergency care*.* *The Journal of Trauma*. **14**(3), 187-196 (1974).

4. Cohen, S., Kamarck, T., and Mermelstein, R., A global measure of perceived stress*.* *J. Health Soc. Behav.*, 385-396 (1983).

5. Mundt, J.C., Marks, I.M., Shear, M.K., and Greist, J.M.H., The Work and Social Adjustment Scale: a simple measure of impairment in functioning*.* *Br. J. Psychiatry*. **180**, 461-464 (2002).

6. Moritz, S., Hormann, C.C., Schroder, J., Berger, T., Jacob, G.A., Meyer, B., et al., Beyond words: Sensory properties of depressive thoughts*.* *Cognition and Emotion*. **28**(6), 1047-1056 (2014).

7. Sheehan, D.V., Lecrubier, Y., Sheehan, K.H., Janavs, J., Weiller, E., Keskiner, A., et al., The validity of the Mini International Neuropsychiatric Interview (MINI) according to the SCID-P and its reliability*.* *Eur. Psychiatry*. **12**(5), 232-241 (1997).

8. Kanstrup, M., Rudman, A., Göransson, K., Andersson, E., Olofsdotter Lauri, K., Denlert, E., Sunnergård, L., Bragesjö, M., Andersson, E., Iyadurai, L., & Holmes, E.A, *Reaching people soon after a traumatic event: Exploring the feasibility of delivering a brief behavioural intervention in the emergency department to prevent intrusive memories of trauma*. under review.

9. Kessler, H., Schmidt, A.-C., James, E.L., Blackwell, S.E., von Rauchhaupt, M., Harren, K., et al., Visuospatial computer game play after memory reminder delivered three days after a traumatic film reduces the number of intrusive memories of the experimental trauma*.* *J. Behav. Ther. Exp. Psychiatry*. 101454 (2019).

10. Visser, R.M., Lau-Zhu, A., Henson, R.N., and Holmes, E.A., Multiple memory systems, multiple time points: how science can inform treatment to control the expression of unwanted emotional memories*.* *Philos. Trans. R. Soc. Lond. B Biol. Sci.* **373**(1742) (2018).

11. Sveriges Radio AB. *Sveriges Radio*. [20.3.4 (5599)] 2018.

12. Iyadurai, L., Blackwell, S.B., Meiser-Stedman, R., Watson, P.C., Bonsall, M.B., Geddes, J.R., et al., Preventing intrusive memories after trauma via a brief intervention involving Tetris computer game play in the emergency department: a proof-of-concept randomized controlled trial*.* *Mol. Psychiatry*. **23**, 674-682 (2018).

13. Weiss, D.S. and Marmar, C.R., *The Impact of Event Scale - Revised*, in *Assessing psychological trauma and PTSD: A handbook for practitioners*, J.P. Wilson and T.M. Keane, Editors. 1997, Guilford Press: New York. p. 399-411.

1. See Figure 1 [↑](#footnote-ref-2)
2. See Figure 1 [↑](#footnote-ref-3)
3. See Figure 1 [↑](#footnote-ref-4)
4. Other reasons patient was screened out before being approached are presented in order of inclusion/exclusion criteria (see Clinical Trials Registry, number NCT03509792) [↑](#footnote-ref-5)
5. In addition to “Can be seen in the emergency department within 6 hours after the traumatic event”, this inclusion criterion was added on 8/5/2019 to also include patients presenting later to the emergency department if still within 72 hours of the event. [↑](#footnote-ref-6)
6. Reasons patient was excluded during eligibility assessment after being approached are presented in order of inclusion/exclusion criteria (see Clinical Trials Registry, number NCT03509792). [↑](#footnote-ref-7)
7. I.e., 12.2% of approached patients [↑](#footnote-ref-8)
8. Section H of MINI 7.0.0 for PTSD consists of a minimum of 1 and maximum of 19 items. For each participant, the number of “yes” responses to each of these 19 items was summed up to compute a total score, ranging from 0 to 19. Higher scores indicate worse symptoms. [↑](#footnote-ref-9)
