# Supplementary figures and images for: Reducing intrusive memories after trauma via a brief cognitive task intervention in the hospital emergency department: an exploratory pilot randomised controlled trial
Source: Transl Psychiatry. 2021 Jan 11;11:30. doi: 10.1038/s41398-020-01124-6 (PMC7798383; doi:10.1038/s41398-020-01124-6)

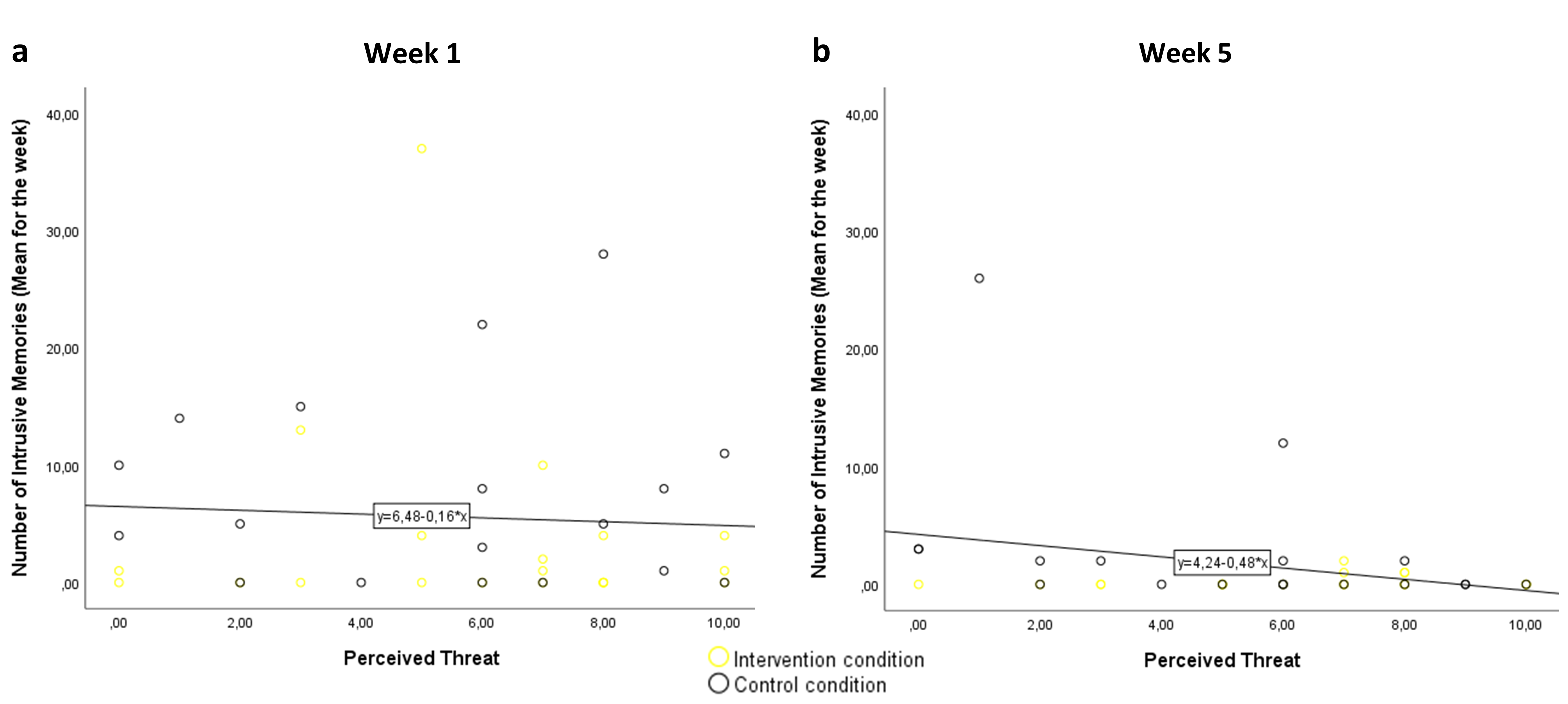

Supplement: Supplementary file 2 — Supplementary Figure 1 [file 41398_2020_1124_MOESM2_ESM.tif]
